# Supplementary material for: Cross-Dataset Single-Cell Analysis Identifies Temporal Alterations in Cell Populations of Primary Pancreatic Tumor and Liver Metastasis
Source: Cancers (Basel). 2023 Apr 21;15(8):2396. doi: 10.3390/cancers15082396 (PMC10137114; doi:10.3390/cancers15082396)

Supplementary Figure S1

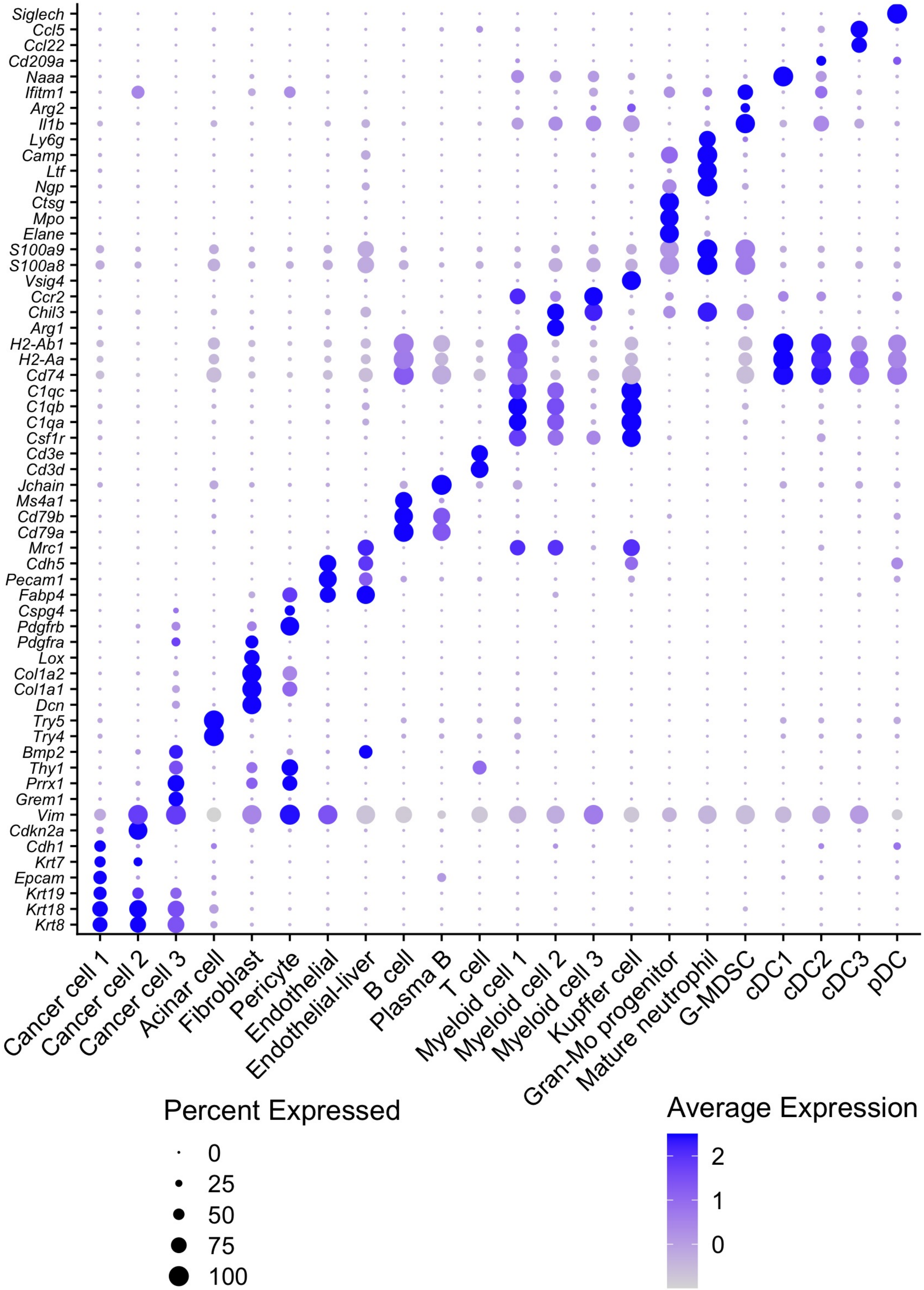

Supplementary Figure S2

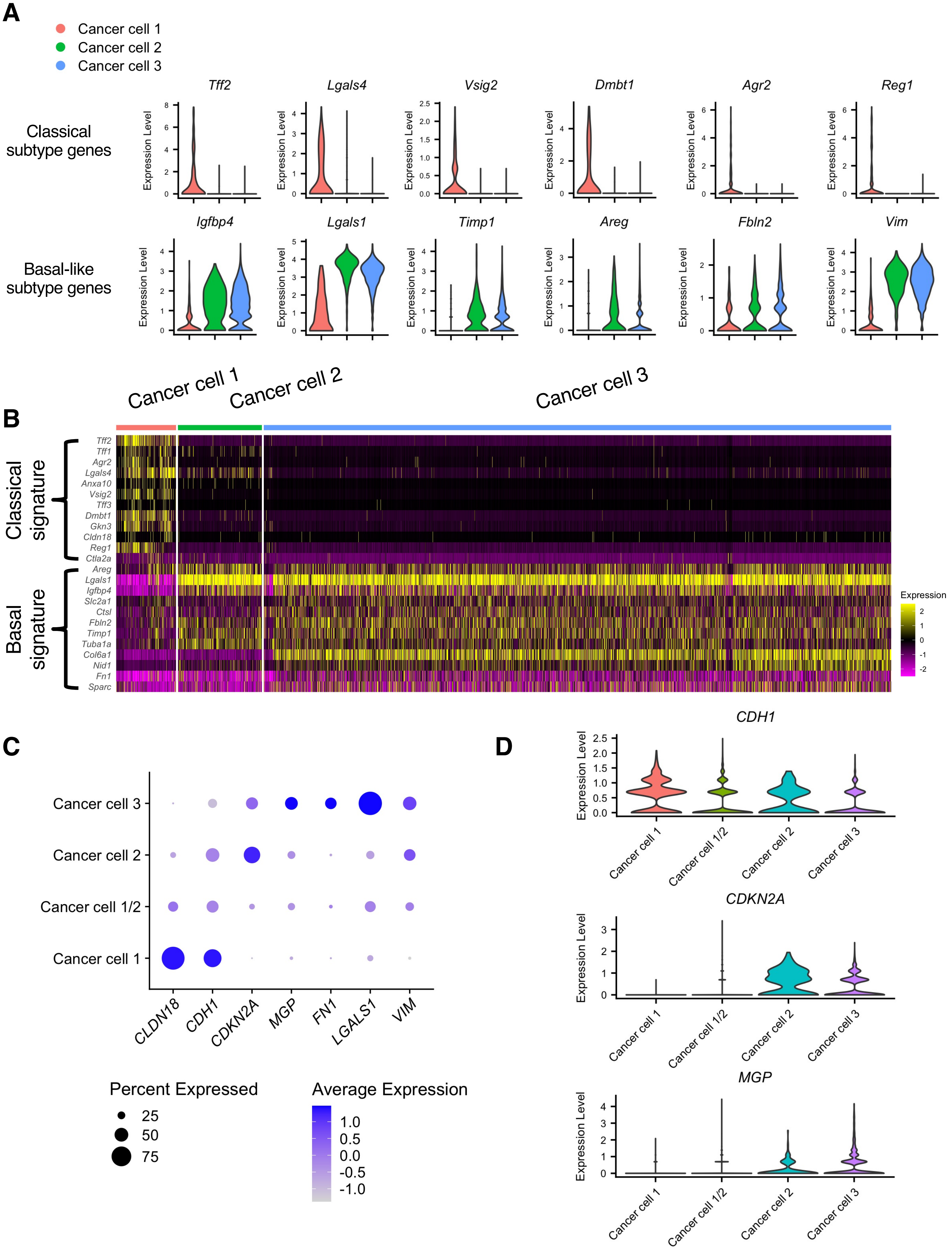

Supplementary Figure S3

A

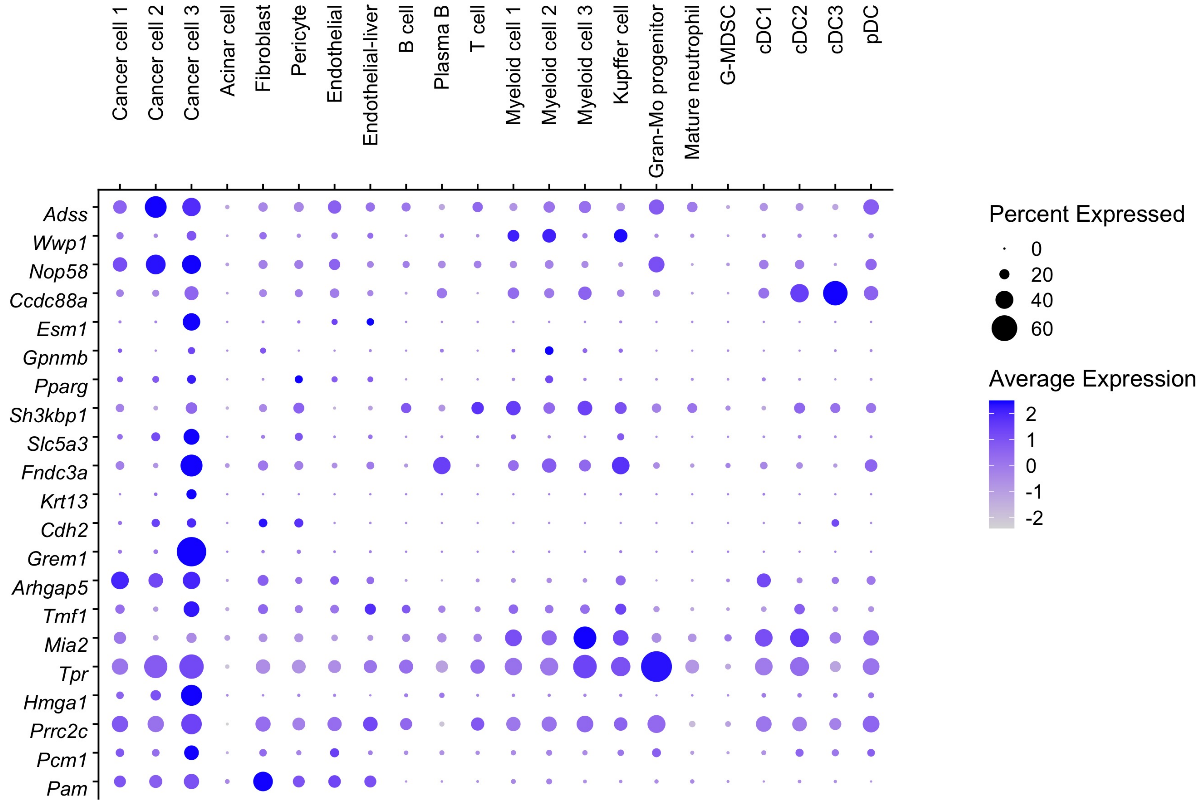

B

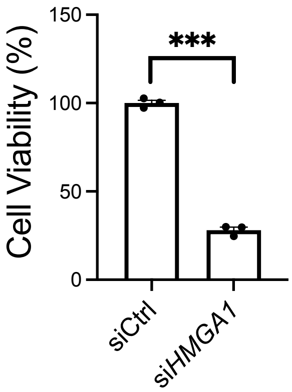

Supplementary Figure S4

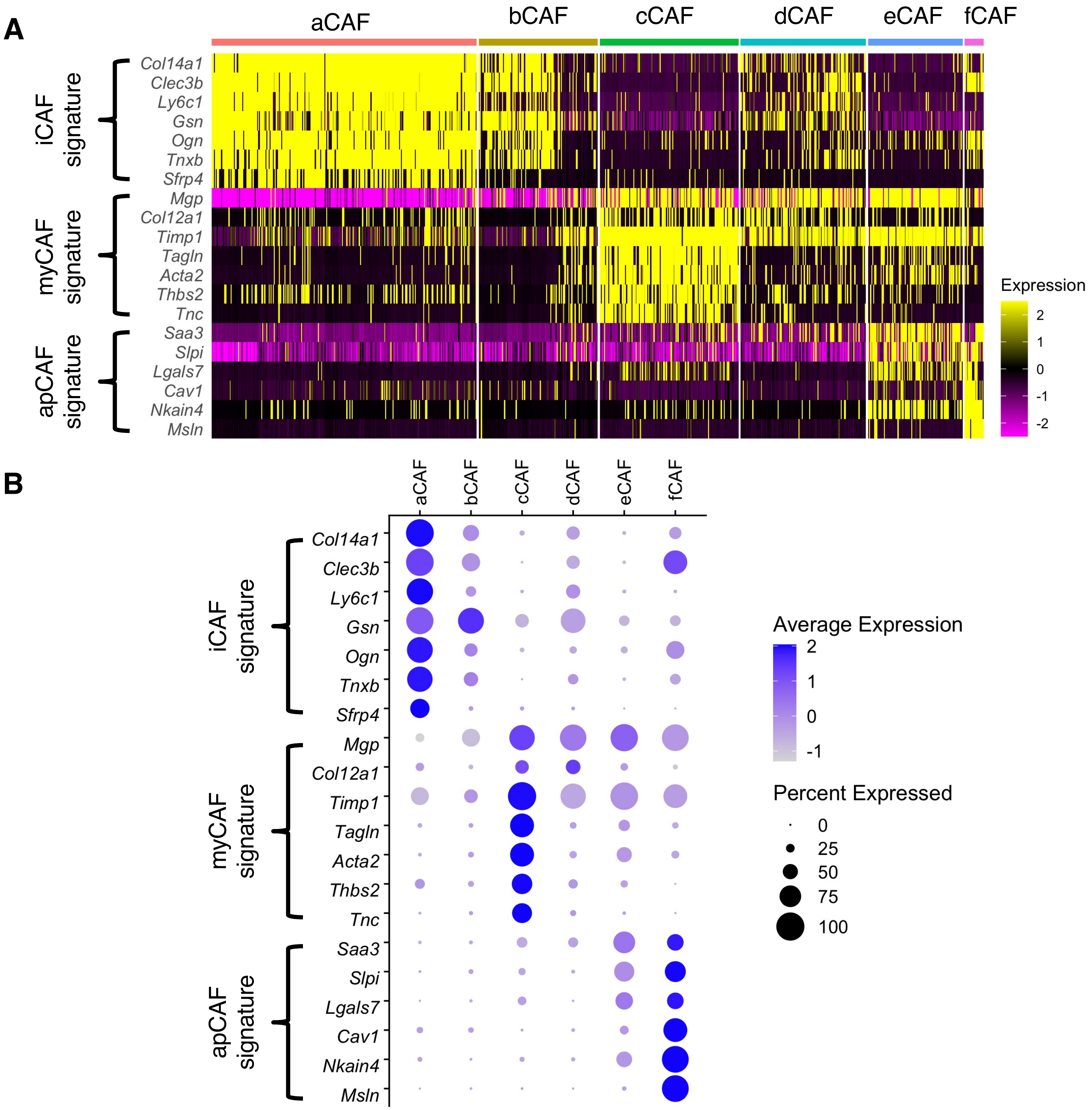

Supplementary Figure S5

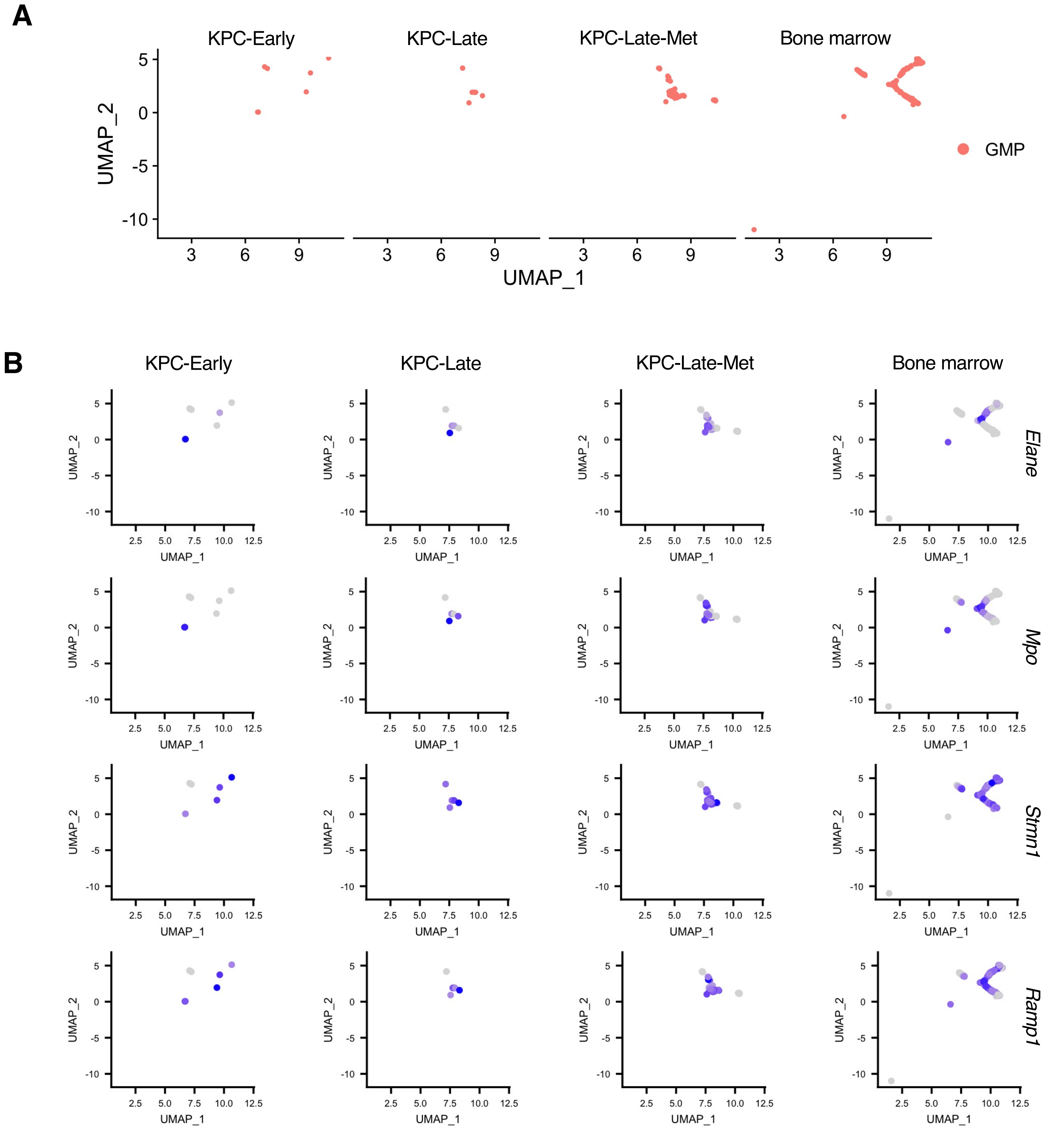

Supplement: Supplementary file 1 [file cancers-15-02396-s001.zip › Supplementary Figures.pdf]
